# Woman: Her Diseases and Remedies

**Published:** 1854-07

**Authors:** 


ART. VI.— *Woman: her Diseases and Remedies.* A Series of Letters to his Class. By CHARLES D. MEIGS, M. D., Prof. of Midwifery, etc., etc. Third Edition, revised and enlarged. Philadelphia: Blanchard & Lea. 1854.

This, as the title indicates, is the third edition of a work, which, to judge from the number of imprints, must have enjoyed a good measure of popularity. Probably most of our readers have perused it, and do not need our dictum relative to its value. But we feel called upon to express an opinion relative to certain of its peculiarities.

The volume is made up in the form of letters to his class. Written to students, its style is familiar, patronizing, and full of those little endearments which occupants of professorial chairs are notoriously apt to lavish upon the disciples of their school. Each letter is from their "affectionate friend," and the series closes with the usual severe paroxysm of grief at parting, to which the tribe of professors are subject, toward the close of February, each year. Perhaps this is all in very good taste. Certainly Dr. Meigs is not peculiar in it. He is only one of the many sufferers from a yearly epidemic.

But this book is published and sent out to the medical world, to educated, professional equals. Does it not, to them, savor somewhat of the pedagogue? Is there not a kind of *me-ipse dixit* about it, against which the sense revolts.

We are aware that the peculiar form of this work was chosen to give a sort of abandon, which should relieve it from that dry, uninteresting character, attributed to medical works in general. Accordingly it is decorated with the flowers of rhetoric; it abounds in quotations from the poets; it evinces great familiarity with metaphysical study; it adopts the absurd and pompous phraseology peculiar to the admirers of Thomas Carlyle, and which was epidemic among a host of imitators at the time these letters were written. If we were to personify Dr. Meigs from his style, we should call him a literary dandy.

We are but too conscious that many medical books are utterly unreadable. But if we would avoid the solemn owlishness of these, is it necessary to assume the mincing manner of the popinjay? Let the reader turn to the pages of Watson for a model of medical style. The physician who finds his lectures dry, or uninteresting, should attribute the fact to his own lack of all medical scholarship. They have an easy elocution; great grace of composition, are filled with happy illustration, and classical adornment; and yet all these beauties are subservient to a certain homely, straight foward purpose *to teach.* And here lies the difference between Watson, and our author. The one adorns that he may teach, the better, and more clearly. The other seems to teach for the purpose of showing off the ornaments of style. A simple fact, a very matter-of-fact amenorrhœa, for instance, is made the peg on which to hang an almost endless disquisition on poetry, art, metaphysics, religion, woman's sphere, and, last and least, menstruation.

Sundry chapters of conversation with patients are given, with the declared intention of instructing the innocent and bashful young gentlemen of the Jefferson school, in the manner of addressing their feminine patients. Of these the most conspicuous is a long, and very spicy interview with Miss Helen Blanque, of No. — Chestnut St. He would be a most verdant and unsophisticated young doctor, who would venture to imitate this model. It is our own serious conviction, that in dealing with the scruples of female delicacy, a short interview is the most pleasant to the lady involved; that the fewer allusions to personal beauty, "budding bosoms," "pouting lips," and "Hebe forms," the fewer blushes will there be, and the less sacrifice of modesty.

Neither have we any sympathy with that overplus of delicacy described on page 447–8. The patient, "a very delicate young lady," was allowed to flow immoderately for many hours, rather than resort to the tampon. We quote. "You will think it strange that I passed the *whole night,* until daybreak, in that apartment, allowing the child to faint again and again; opening the windows; using the fan; applying iced vinegar cloths to the hypogastrium and thighs; administering opium, sulphuric acid, and rose-infusion; taking the pillows occasionally from under the head, and vainly endeavoring to re-assure the mother who repeatedly entreated me to use the tampon; but I would not."

Dr. Meigs is right. We do think it *very* strange. He was finally compelled to use the tampon. There was no salvation of delicacy to compensate for a night of dreadful anxiety; and loss of blood so great as to make a spinal irritation, or a prolonged convalescence, almost a certainty. Whenever we meet with such overstrained, and sickly false modesty, we are forcibly reminded of Prof. Ackley's stout assertion, that "It was impeaching the wisdom of the Almighty to say that a child could be born in an indecent manner." Another noteworthy feature in these letters is the bare, single mention which the speculum receives. Previously to the first edition, Dr. Meigs had stood prominently forth as an opponent of this instrument. At the time of writing it, he was gently sliding from the elevation of that immaculate modesty which preferred death to profanation, toward the common, and extremely vulgar level of ocular inspection. In his late report on the diseases of the uterus, he occupies a position decidedly in favor of the instrument. We are glad to see this. His opposition to the speculum, like other faults of the book under criticism, originated in an entirely false conception of the duties of the physician as opposed to female delicacy. He is now on the true ground, and seems inclined to make up lost time in this regard.

The ideas of pathology and treatment presented to us, are not as open to criticism as the style in which they are clothed. Dr. Meigs, without claim to discovery, or great original talent, is yet a thorough scholar, and deeply versed in his specialty. When he occasionally throws off the fetters of a stilted oratory, he is a good, common sense, and practical teacher.

We may as well confess, finally, that "Meigs on Woman, her Diseases and her Remedies," is what it purports to be—a very readable book. We trust, however, that it may have few imitators. Most attempts at "doing the genteel," in medical writings, are eminent failures. All are failures in one sense. They convey but a small modicum of information, in a deal of diligent reading.

For sale by Miller, Orton & Mulligan. H.

